# *On the* Symptoms *and* Treatment *of the* Fever *at* Gibraltar

**Published:** 1805-09-01

**Authors:** Robert Thomas

**Affiliations:** Guilford


### *On the* Symptoms *and* Treatment *of the* Fever *at* Gibraltar; *communicated by* Robert Thomas, M. D. *of* Guilford.

THE disease is the Typhus gravior, or jail, or malignant fever.

Great preceding heats, a numerous population in confined situations, and small houses, inattention to proper cleanliness, and a free ventilation, a neglect of separating the infected from the healthy, and of cutting off all unnecessary communication between them, and no efficacious means having been early resorted to for subduing or eradicating the contagion, this fever has now acquired a degree of virulence and malignity equal to the plague itself.

This disorder is highly contagious; as all fevers of the typhoid kind are universally admitted to be capable of being propagated from one individual to another, either by contact or by inhaling the effluvia arising from the body of a diseased person, or from linen clothes and other articles, strongly impregnated with their miasma.

Universal weariness, faintness, great depression of strength, severe pains in the back and head, particularly in the forehead and sockets of the eyes; rigors succeeded by universal heat; thirst, a parched tongue, somewhat covered with a brown fur; nausea, with now and then a vomiting of bilious matter; costiveness, laborious respiration, accompanied accompanied with deep sighing; a small but quick pulse, of from 100 to 140 in a minute; slight wanderings, incoherency and coma, are the most usual symptoms attendant on this fever; but in its very advanced state, those of putrescency are observable. In some cases, the patient will die on the fourth day.

By the following mode of treatment, I have never lost a patient, where my advice has been applied for early.

If the person is incommoded by nausea or vomiting, on his seizure with this fever, I would recommend the contents of the stomach to be evacuated, by directing him to drink a few cups of a strong infusion of camomile flowers, in preference to his taking an emetic, either of ipecacuanha or tartarized antimony; which seem inadviseable, from the great irritability of this organ, which is apt spontaneously to ensue. If nausea does not prevail, then the first step to be adopted, should be to clear the bowels of all feculent matter, by a sufficient dose of calomel; the operation of which may be rendered more certain and quick, by an addition of a few grains of coloquintida.

The bowels being cleansed, I advise an immediate use of the muriatic acid. To adults, we may give ten or twelve drops at first, which dose should be repeated every four hours, guarded by four or five drops of the tincture of opium, to prevent the acid from acting unpleasantly on the stomach and intestines. By degrees, we may increase the quantity of the acid in each dose to eighteen or twenty drops, employing as a vehicle, about an ounce and a half of a strong infusion of columbo: the effect of mineral acids, but more particularly the muriatic, in all fevers of a malignant nature, is truly great; and from employing it in all such cases, my practice has been marked with the most decided success. To increase the antiseptic effect of this medicine, as well as to obviate debility, I always recommend a quantity of wine, proportionable to the age of the patient, and the exigency of the case, to be administered at the same time. Bleeding should never be used; as the Spanish physicians, at Malaga, sacrificed most of their patients by it.

At a very early period of the disease, when the rigors have ceased, and the skin is become dry and hot, I strongly advise a general affusion of the whole body with cold water, as recommended by Dr. Currie, of Liverpool; and since practised by myself, and many other physicians, in innumerable instances, with the happiest effect. This remedy may be repeated twice or thrice in the course of the the twenty-four hours; but it should be at those periods when there is no great sense of chilliness present, when the heat of the body is steadily above the natural, and when there is no general or profuse perspiration. Where either the delicacy of the system, the apprehensions of the patient, or the prejudices of the bye-standers, prevent the employment of cold affusion, we must be content to substitute tepid affusion for the more powerful remedy. The earlier in the disease that cold affusion is employed, the more likely will it be to bring it to a favourable termination, should it fail in arresting its progress wholly.

Throughout the whole course of this malignant disorder, the bowels ought to be kept open, by administering, as occasion may require, the laxative of calomel, before recommended; but I do not think that mercury should be given in sufficient doses to excite any degree of salivation, as has been practised in the yellow fever. Should further experience, however, sanction this mode of proceeding, the process will not interfere with the employment of tepid affusion at the same time, although it might with that of the more powerful remedy.

As I am induced to suppose, that a high degree of delirium does not often attend on the fever in question; and that the patient is rather incommoded by muttering and slight wanderings, I do not hesitate to advise the giving a dose of opium, proportioned to the age of the person, every evening at an early hour; which practice I generally adopt in all fevers of the typhoid class, and with very good effect.

Having pointed out the means which I think most likely to procure a favourable termination of the disease, I beg leave to add a few monitory cautions for suppressing its further propagation, and destroying its contagion. To answer the first of these intentions, it will be necessary to keep the mind cheerful, and as free from all apprehensions and anxiety as possible; and carefully to avoid intemperance, sensuality, great fatigue, profuse evacuations, a poor vapid diet, or whatever else may tend to produce debility. By strengthening the bodies of men, it is supposed they will thereby be enabled to resist contagion the better; I would, therefore, recommend cold bathing every morning, with two or three doses daily of some tonic medicine; such as the Peruvian bark. If wine is used at all, it should be sparingly; the less perhaps the better.

Nurses, and medical attendants, who are immediately exposed to the contagion, should be careful to come into immediate immediate contact with the diseased as seldom as possible; and they ought never to inhale the breath of the sick, nor place themselves in such a direction, as that a stream of air can waft the miasms or effluvia towards them. Possibly, it might be advantageous to anoint the hands with sweet oil, as has been practised, it is said, in the plague, with much advantage.

For the purpose of destroying the contagion, the sick should be removed to Lazarettos; and these must be guarded, so as to cut off all unnecessary communications with those in health. The atmosphere surrounding the infected, should be purified as much as possible, by a strict attention to cleanliness, a free ventilation, and frequent fumigations with the nitrous or muriatic acid, in the form of gas. All substances capable of being impregnated with the effluvia, and of vitiating the atmosphere, should be speedily removed from the apartments of the sick, to situations where the healthy cannot suffer by them, and where they will be made to undergo proper purification.

It does not signify which of the acids we employ, as they are both equally efficacious in destroying every species of contagion. If a preference be given to the muriatic, place a saucer, or any other earthen vessel, containing about half a pound of common salt, in the apartment of the sick, and pour over it, from time to time, a sufficient quantity of vitriolic acid, to moisten the whole of the salt. If the nitrous is preferred, put half an ounce of vitriolic acid into a cup, saucer, or glass, and add, from time to time, some nitre reduced to powder. In rooms from fifteen to twenty feet in dimensions, one vessel will be sufficient; but in larger ones, two or more will be requisite; and when the air is foul, and peculiarly offensive, it will be adviseable to apply a slight degree of heat under the vessels, in order to extricate a larger quantity of vapour.

Europeans of a full plethoric habit of body, who may be obliged to go out to Gibraltar during the prevalence of this malignant fever, will act prudently in taking now and then, during the passage, some cooling laxative medicine; and in undergoing a slight mercurial course, so as to produce an alterative effect. The same plan was found to be a good preventive against any attack of the yellow fever.

*July* 13, 1805.

